# Radiological and surgical aspects of round window visibility during cochlear implantation: a retrospective analysis

**DOI:** 10.1007/s00405-021-06611-0

**Published:** 2021-01-20

**Authors:** Saad Jwair, Job J. M. van Eijden, Esther E. Blijleven, Jan Willem Dankbaar, Hans G. X. M. Thomeer

**Affiliations:** 1 Department of Otorhinolaryngology and Head & Neck Surgery, University Medical Center Utrecht, Utrecht University, Heidelberglaan 100, P.O. Box 85500, 3508 GA Utrecht, The Netherlands; 2 UMC Utrecht Brain Center, Utrecht University, Utrecht, The Netherlands; 3 Department of Radiology, University Medical Center Utrecht, PO Box 85500, 3508 GA Utrecht, The Netherlands

**Keywords:** Auditory prostheses, Cochlear implant, Round window, Facial nerve, Chorda tympani nerve

## Abstract

**Purpose:**

The round window approach has become the most preferred option for cochlear implant (CI) insertion, however, sometimes it may not be possible due to the (in)visibility of the round window membrane (RWM). We addressed the prevalence, consequences and indicators of difficult detection of the RWM in cochlear implant surgery.

**Methods:**

This study retrospectively analysed the operative reports and preoperative high resolution axial-computed tomography (CT) scans of a consecutive cohort of patients who underwent a CI insertion. The main outcomes were surgical outcomes of the RW approach, and assessment of radiological markers.

**Results:**

The operative reports showed that RWM insertion was feasible in 151 out of 153 patients. In 18% of the patients the RWM was difficult to visualize. All these patients had at least one intraoperative event. The chorda tympani nerve (CTN) or posterior canal wall was affected in 8% of the 153 patients and the fallopian canal in 6%. These patients had a facial-chorda tympani nerve distance on the CT scan that was considerably smaller than normal patients (1.5 mm vs 2.3 mm). In addition, a prediction line towards the anterolateral side of the RWM was found to be more prevalent in these patients’ CT scans (sensitivity 81%, specificity 63%).

**Conclusion:**

The RW approach is feasible in almost all patients undergoing CI surgery. Difficult visualisation of the RWM seems to lead to at least one intraoperative event. Radiological measures showed that these patients had a smaller facial recess and a more anteriorly placed facial nerve, which can be used to better plan a safe insertion approach.

## Introduction

Cochlear implants (CIs) provide a solution for patients of all ages with severely impaired hearing. The classical surgical method of implantation is performed by way of a retro-auricular approach with a mastoidectomy-facial recess technique, followed by a CI insertion via either the round window membrane (RWM) or an anteroinferiorly (relative to the RWM) placed cochleostomy [1, 2]. This surgical method is standard care in most CI centres worldwide [3]. The RW approach is nowadays normally preferred over a cochleostomy because it might be less traumatic [1, 4].

Although the RW approach is widely adopted, only few studies reported its feasibility and complications [3, 5]. The RW approach is not always possible, presumably because of the sometimes difficult visualisation of the RWM [2, 3, 6]. Intraoperatively, trying to improve visibility of the RWM can lead to an increased chance of intentional or unintentional damage to important structures like the chorda tympani nerve (CTN), the fallopian canal, posterior canal wall or tympanic membrane. Although this damage does not necessarily lead to postoperative complications, it is preferred to leave these structures intact [7]. To avoid these situations, it might be beneficial to assess the RWM visibility before surgery.

In current medical practice RWM visibility is not assessed beforehand. A preoperative high resolution computed tomography (HRCT) is used to assess medical contraindications for a RW approach (e.g. otosclerosis or cochlear malformations) [8]. In addition, surgeons use this scan to be adequately prepared for surgery, by assessing important surgical landmarks such as the sigmoid sinus, incus and lateral semicircular canal [8, 9]. Previous studies have shown that these scans can also be used for investigation of the RWM visibility [10–13].

For this study we outlined two goals regarding cochlear implantation surgery: (1) to identify the feasibility of the RW approach in our adult population, and (2) to assess the prevalence, consequences and radiological markers of difficult RWM visibility.

## Materials and methods

### Study design

The operative reports and preoperative HRCT scans of a cohort of adult patients that received a CI at our tertiary referral centre between January 2015 and March 2020 were retrospectively examined. These patients were consecutively operated by one surgeon. The data were collected from the patient files. The eligibility criteria were as follows: (1) age ≥ 18 years, (2) no inner ear deformities, (3) primary cochlear implantation, (4) no prior mastoid or middle ear surgery on the implanted side, (5) no signs of (chronic) otomastoiditis, (6) patent RWM and scala tympani (ST) of implanted side on preoperative HRCT scan. The first five items were assessed with the operative and medical report data. If discussion on the eligibility criteria was encountered, consensus was obtained between the authors.

### Operative report

The operative report of every patient of the database was evaluated by two investigators (SJ and JvE). The following variables were extracted: age, gender, medical diagnosis, side of implantation, type of middle ear and insertion approach, mastoid pneumatisation, view of RWM (easy or difficult), facial recess size (normal or small), other notable issues (e.g. overhanging posterior wall or bulging jugular bulb), and lastly intraoperative events (e.g. lesions of the CTN, posterior wall, facial nerve (FN) and fallopian canal). In addition, the postoperative medical reports of cases with an intraoperative event involving the FN or CTN were reviewed for related complaints (e.g. tongue sensitization or face paralysis).

### High resolution CT scan

High resolution temporal bone images (axial and coronal plane reconstructions) with a slice thickness of 1.0 mm were obtained using a Siemens-force CT scanner at 120 kV and 150mAs or a Philips scanner at 120 kV 300mAs. Two investigators (SJ and JvE) analysed and gathered the HRCT scans. These investigators were not involved in any of the surgeries, and were blinded for the operative findings during the analysis of the HRCT scans. Beforehand, the investigators were trained by an ENT surgeon (HT) and neuroradiologist (JWD) in the analysis of the mastoid, with an extra focus on the course of the FN and CTN.

The CTN was identified by three points:

1. Origin of the FN at mastoid tip
2. Mastoidal course until tympanic annulus (bony rim of the tympanic membrane)
3. Re-appearing again at the anterior wall of the middle ear cavity and entering the petrotympanic fissure.

**Fig. 1:**
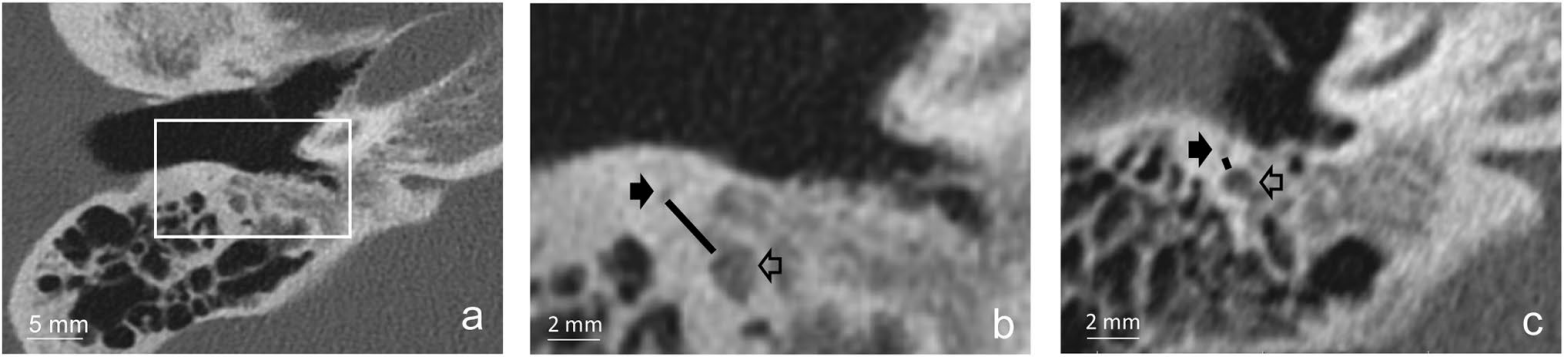
**a** Overview of the preoperative axial high resolution CT scan of the right temporal bone. **b** Magnification (× 2.5) of the same axial high resolution CT scan of the right temporal bone. Black arrow depicts the chorda tympani nerve (CTN), and the unfilled arrow the facial nerve (FN). The line between these two nerves is the FN-CTN distance. This case had a FN-CTN distance of 2.9 mm**. c** Axial high resolution CT scan of the right temporal bone of another patient. This case had a small FN-CTN distance of 0.6 mm

The authors drew a line between the FN and the CTN on the axial HRCT scan, see Fig. 1. The measurement of the FN-CTN distance was defined by the shortest distance (inner margin) between two points on the axial HRCT reconstructions with the posterior canal wall/mastoid and the middle ear space within the same plane:

1. The CTN, as close as possible to its entry in the middle ear space, but still in the mastoid.
2. FN, at the point of the second genu.

The second last axial HRCT section of the mastoid segment of the CTN, before entering the middle ear space, proved to be the most optimal section to measure the FN-CTN distance. This measurement enabled us to confidently state the near maximal distance of the facial recess opening between the FN and CTN.

**Fig. 2:**
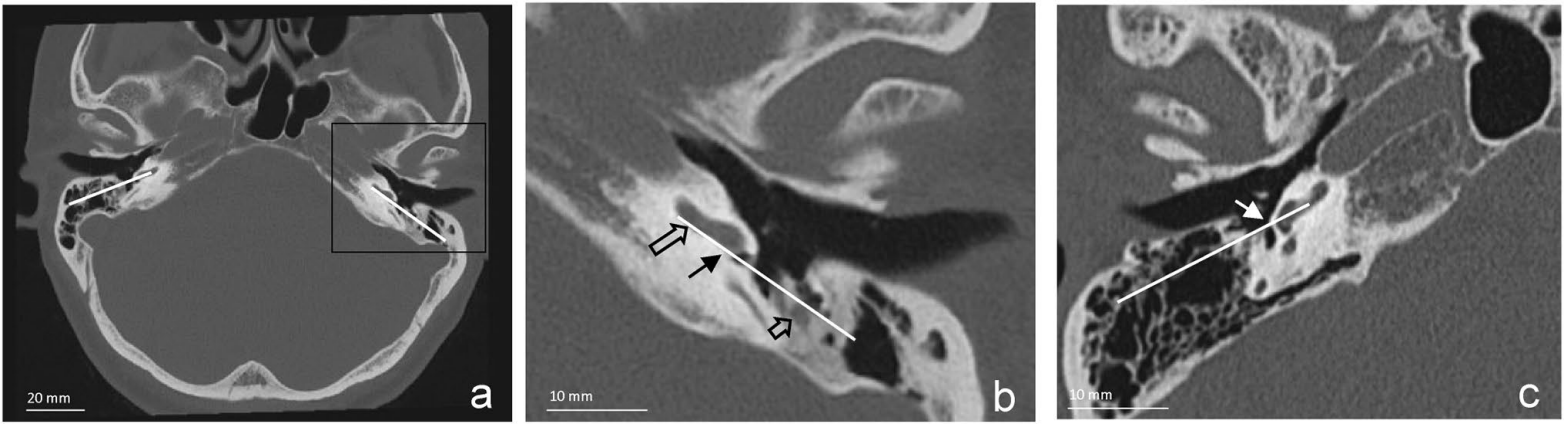
**a** Prediction line drawn on the axial high resolution scan of both temporal bones. **b** Close up view of the prediction line. The prediction line was drawn on the preoperative axial high resolution CT scans between the anterolateral mastoid facial nerve and the lower side of the basal turn of the cochlea. The intersection point lies on the posteromedial side of the round window membrane in this example. Large unfilled arrow = lower side of basal turn, filled black arrow = posteromedial intersection point, small unfilled arrow = facial nerve. **c** Example of an intersection point on the anterolateral side of the RWM. White arrow = anterolateral intersection point

The authors established a second measurement, partly based on a previous study [12], that indicated the anterior position of the FN relative to the RWM. A prediction line was drawn from the anterior part of the mastoid course of the FN on the axial planes, towards the lower side of the basal turn of the cochlea. Subsequently, the intersection point between the RWM and the prediction line is categorized in being either anterolateral or posteromedial, see Fig. 2. All intersection points below the middle of the RWM were classified as posteromedial, and the intersection points above this middle were classified as anterolateral.

### Analysis

Based on the operative reports we established whether the intended RWM insertion was successful. The operative reports were also used to assess the intraoperative visibility of the RWM. Two groups were identified: cases with normal identification of the RWM and cases with difficult visibility of the RWM. Cases with difficult RW niche visibility were also included in the latter group. After excluding all cases with inadequate scans we compared the radiological measurements between the normal and difficult cases. For the second radiological measurement (i.e. prediction line) 20 cases of the normal group, at random, were selected for the comparison analysis. All radiological analyses were done blinded for the operative report and outcomes.

## Results

**Fig. 3:**
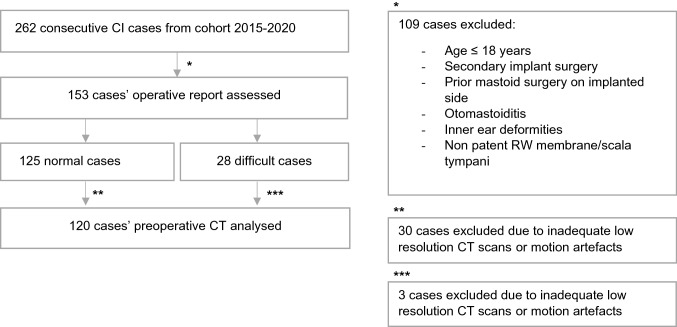
Flow chart of the in- and excluded cases for both the operative report and preoperative computed tomography scan analyses

The patient cohort, January 2015–May 2020, was screened for the in- and exclusion criteria (see Fig. 3). After applying these criteria, 153 cases were included for the operative report analysis. Regarding the HRCT analyses, we had to exclude 33 from 153 cases, 30 from the normal group and 3 from the difficult group, because in those cases the only available scan was of a low-quality CT with an inadequate image resolution or with severe motion artefacts. In total, 120 HRCTs were analysed.

### Operative report

**Fig. 4:**
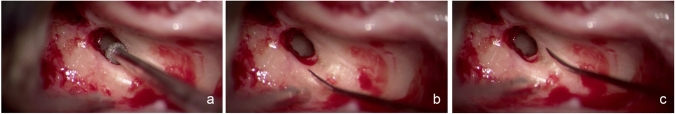
**a** Intraoperative view of the facial recess opening (right ear), a 2 mm burr fits easily in the facial recess opening. **b** Facial nerve is clearly identifiable, with an intact posterior canal wall. **c** Chorda tympani nerve is also clearly identifiable, the round window is seen posteroinferiorly in the facial recess opening

In 151 out of 153 patients (99%) a RWM insertion was realized and successful, the other two patients received a cochleostomy. An example of the intraoperative view is depicted in Fig. 4. That example would classify as a normal case, as the surgeon is able to identify the RWM with intact anatomical borders (i.e. FN, CTN, incus buttress and posterior canal wall). The patient characteristics and intraoperative events are summarized in Table 1.

**Table 1:** Patient characteristics and outcomes, *n* = 153 (%)

Age at implantation, years (SD)	62 (16)
Gender	
Male	80 (52)
Female	73 (48)
Ethnicity	
Native Dutch	149 (97)
Diagnosis	
Progressive bilateral SNHL	151 (99)
Side of implantation	
Right	72 (47)
Left	80 (52)
Bilateral	1 (< 1)
Mastoid pneumatization	
Sclerotic	14 (9)
Type of middle ear approach	
Mastoidectomy-facial recess	153 (100)
Type of insertion approach	
Direct RWM	151 (99)
Cochleostomy	2 (1)
Intraoperative events^a^	
Facial nerve exposure	10 (6)
Chorda tympani nerve lesion	13 (8)
EAM/TM lesion	12 (8)
Other^b^	14 (9)

*EAM* external auditory meatus, *TM* tympanic membrane, *RWM* round window membrane, *SD* standard deviation

^a^Some patients had more than one event; ^b^includes venous bleeding and tegmen tympani lesions

In total, in 28 patients (18% from total), the RWM and niche detection was difficult, mostly due to a small facial recess (26/28). In one case, the posterior canal wall was hindering the surgeons view, while another case had a high riding jugular bulb obstructing the RWM access. Interestingly, all these patients had at least one intraoperative event. The chorda tympani nerve (CTN) was sacrificed in 13 cases (8%), posterior canal wall lesions in 12 cases (8%) and fallopian canal uncovering in 10 cases (6%). The CTN had to be sacrificed to provide adequate visualisation of the RWM and niche. Postoperative medical reports showed no complaints related to the CTN sacrifice (e.g. taste disturbance or tongue sensitization). Furthermore, to improve the visibility through the facial recess opening, a small part of the bony cover of the FN canal had to be removed. No FN weakness direct postoperatively or long term was noted in any case. Finally, no complaints were detected for patients with the partial uncovering of the bony posterior wall of the external auditory canal or tympanic membrane annulus.

The intraoperative findings were evenly spread over time (during the included period of investigation) and side of implantation, indicating no relationship of these findings with either of these factors.

### High resolution CT scan

In total, 120 HRCT scans were analysed for the FN-CTN distance, and divided in two groups based on the operative reports: 95 scans of the normal cases, and 25 scans of the difficult cases. For the prediction line, 20 cases of the normal group, at random, were selected for comparative analysis with the difficult cases. A sclerotic mastoid was seen in 10% of the patients (in concordance with the operative reports), no difference was observed between both groups.

### Facial-chorda tympani nerve distance

The mean FN-CTN distance was 2.2 mm (SD: 0.5, confidence interval 2.12–2.32) for the normal cases (*n* = 95), in contrast, the mean distance was 1.5 mm (SD: 0.4, confidence interval 1.31–1.68) for cases with difficult view of the RWM (*n* = 23), which is a significant difference (*t* test, *p* < 0.001). The FN-CTN distance of ≤ 1.5 mm was applicable for 9 patients (9%) of the normal group, and for 17 patients (74%) of the difficult cases group, resulting in a sensitivity of 65% and a specificity of 93%. Two cases with a difficult view of the RWM were left out of this analysis, because the visibility of the RWM was hindered by other factors than the facial recess opening. The FN-CTN distance was 2.9 mm for one case with an overhanging posterior canal wall, and the other case had a high riding jugular bulb with a FN-CTN distance of 2.2 mm.

### Prediction line

**Fig. 5:**
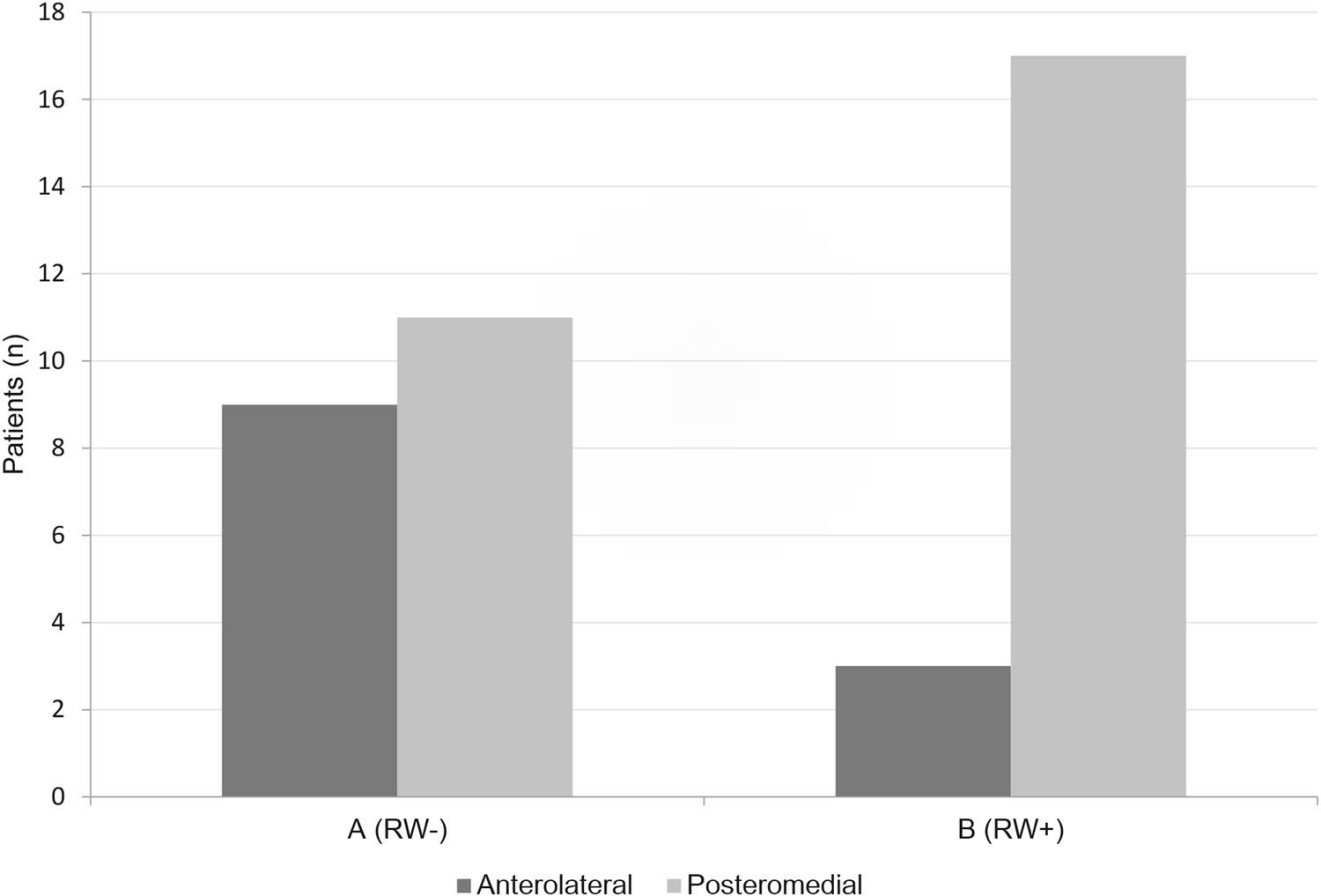
Comparison between group A (difficult visualisation of RW) and B (easy visualisation of the RW) of the intersection point on the RWM of the prediction line. The intersection point was on the posteromedial part of the RW in most patients with a good intraoperative visualisation of the RW. Both groups consisted of 20 patients. *RW* round window

Axial HRCT reconstructions showed that the anterolateral FN and the basal turn of the cochlea could not be reliable identified in 3 out of 23 cases with a difficult view of the RWM and niche. Those cases were therefore excluded, resulting in 20 included cases with difficult view of the RWM. Analysis showed that in group A (difficult cases) 9 out of 20 had an anterolateral intersection point, and 11 out of 20 had a posteromedial intersection point. For group B (normal cases) 3 out of 20 had an anterolateral intersection point, and 17 out of 20 had a posteromedial intersection point. See Fig. 5 for a summary of these results. The sensitivity was 81%, and specificity 63%, with a posteromedial intersection point being favourable for easy or normal detection of the RWM. No differences were observed between both sides within cases.

## Discussion

### Operative report

Our study shows that a direct RW approach is feasible in almost all cases (99%). In addition, the RWM was difficult to visualize in 18% of the cases, usually because of a small facial recess (*n* = 26/28). In 13 cases (8% of total), the CTN had to be sacrificed to visualize the RWM. Clearly, in those cases, the CTN was limiting the viewing angle through the facial recess. In the remaining cases with a narrow facial recess the surgeon presumably succeeded in retaining the CTN, while implanting via the RWM. The retrospective design of this study, however, meant that we were limited to the retrospective operative reports, introducing possible bias. In addition, only crude estimations of the relevant outcomes were possible, i.e., we were only able to discern between easy and difficult cases.

Another study showed that direct RWM insertion is almost always possible, however, without reporting intraoperative events to important landmarks [14]. In contrast, other studies indicate that a direct RWM insertion is not always possible [3, 5, 6, 12, 15]. In these studies, the rate of unsuccessful direct RWM insertion ranges between 7 and 15%, often necessitating a conventional cochleostomy.

The surgical approach in our study involved maximal exposure of the facial recess, while preserving the integrity of the FN (fallopian canal), CTN, posterior canal wall and bony tympanic annulus whenever possible, followed by drilling of the bony overhang of the RW niche to expose the RWM. The posterior canal wall was often thinned as much as possible. Subsequently, if needed, the CTN was sacrificed to visualize the RWM, potentially explaining the higher success rate of this study.

Other causes that can obscure the RWM visibility have been described in previous studies, such as a ‘high riding’ jugular bulb or an overhanging posterior canal wall [6, 12, 16, 17]. In this study, there was one case with an overhanging posterior wall, and one with a high riding jugular bulb. In our cohort and in previous studies, the obscuration of the facial recess opening by the posterior wall or sigmoid sinus and jugular bulb is a rare phenomenon (< 1%) [14, 18]. Some surgeons advocate in cases of an overhanging posterior wall, to “green stick fracture” the posterior wall medially (just lateral from the FN and push it forward) providing improved exposition of the RWM, access to the middle ear and perform implantation of the electrode array; then replace the (partly mobile) canal wall to its previous position where bone will regrow.

Lastly, we identified no cases with postoperative complaints related to the CTN or FN, although patients with CTN lesions only mention their taste disturbances postoperatively if they are asked for it [19]. Other studies also showed that FN paralysis occurs infrequently (< 1%) following cochlear implantation procedure with a mastoidectomy-facial recess approach [7, 20]. In contrast, postoperative complications related to the CTN seem to occur more often (> 2%), although rates vary widely between studies [7, 19].

### Radiological measurements

#### Facial-chorda tympani nerve distance

Comparison of the radiological measurements of the FN-CTN distance between cases with normal and difficult visibility of RWM showed a smaller FN-CTN distance (difference of 0.7 mm) for the cases with difficult visibility. Therefore, the FN-CTN measurements corresponded to the subjective outcome of the operative reports (i.e. small facial recess). These results show that the FN-CTN distance indeed provides a realistic estimate of the size of the ‘window’ to the middle ear structures [16]. A previous study also showed that the FN-CTN distance in the mastoid is important for the viewing angle through the facial recess opening [18]. Two other studies in adults showed no effect of the facial recess width on the visibility of the RW [10, 12]. These studies, however, measured the width of the facial recess using the posterior canal wall and FN. A correct facial recess opening, in our opinion, is the distance between the FN and CTN. By opting for the posterior wall, the mentioned study could have measured a facial recess width that was larger than what was actually possible intraoperatively.

#### Prediction line

Our study shows that the prediction line between the basal turn of the cochlea and the FN can be important in indicating the visibility of the RWM intraoperatively. A different study showed that the RWM visibility, classified into three types (invisible/nearly invisible, partially visible, fully visible), was predicted by a line drawn parallel to the external auditory canal and the FN [10]. The basal turn of the cochlea was in our experience more reliably and easier determined than a line parallel to the canal.

Previous studies have shown that the course of the FN can be highly heterogeneous, and might play a role in RWM visibility. In addition, the angle of rotation of the RWM plays an important role as well. These two aspects both heavily influence the outcome (anterolateral vs posteromedial intersection point) of our prediction line, confirming indeed their importance in determining the viewing angle of the RWM.

### Clinical perspectives

In this study, a RWM insertion approach was chosen for all patients if the ST and RWM were patent on the preoperative CT scan. The CTN was sacrificed if the RWM was difficult to recognize, achieving a high rate of direct RWM insertions. Other studies chose in such cases to convert the RWM approach to a conventional cochleostomy [6, 10]. It is unclear which of these two options is the best choice for patients when the RWM is not or barely visible. On the one hand, opting for a conversion of insertion access to the cochlea by a conventional cochleostomy has its own potential downsides. An important rationale for direct RWM insertion, is that the RWM forms a natural gateway to the ST of the cochlea thereby preserving as much as possible the cochlear anatomy and inner ear microstructures. A cochleostomy also might lead to increased chance of translocation of the electrode array, or missing the ST altogether, leading to a direct scala vestibuli insertion, potentially negatively impacting the overall hearing outcomes of the CI user [21, 22]. Some surgeons, however, advocate that the vector of insertion angle might be more parallel and in line with the ST direction in the basal turn in contrast with RWM insertion. On the other hand, sacrificing the CTN can lead to symptoms such as a dry mouth and taste disorders [19, 23]. However, these symptoms might not always lead to persistent and troublesome complaints, and the recovery rate can be as high as 79% after CTN lesion [19]. Probably the rate of postoperative complaints related to the CTN is underestimated, because most patients with CTN lesions only mention their taste disturbances postoperatively if they are asked for it.The high recovery rate of the CTN can be potentially explained by improved functioning of the ipsilateral glossopharyngeal nerve, re-innervation via contralateral or ipsilateral glossopharyngeal nerve and CTN, and by subjective adaption of patients [19, 23]. Of course, both these options’ advantages and disadvantages should be weighed against the specific clinical characteristics of the patient, e.g., in a patient with preoperative taste disturbances sacrificing the CTN would be contraindicated.

## Conclusion

The RW approach for cochlear implantation seems feasible for most patients in our population. Difficult visualization of the RWM seems to lead to at least one intraoperative event in patients. The intraoperative events were iatrogenic damage of the CTN, (minor) exposure of the FN epineurium and lesions of the posterior canal wall. These patients had on the preoperative HRCT scan a smaller facial recess, and a more anterior position of the FN relative to the RW niche. These factors can be used to plan an insertion approach in cochlear implantation procedures, potentially leading to less iatrogenic damage of especially the CTN.

## Data Availability

Data sharing, including full protocol, participant datasets and statistical codes will be considered upon reasonable request.
